# Unrecognized risks and challenges of water as a major focus of COVID-19 spread

**DOI:** 10.7189/jogh.11.03016

**Published:** 2021-01-16

**Authors:** Amir Khorram-Manesh, Krzysztof Goniewicz, Frederick M Burkle

**Affiliations:** 1 Institute of Clinical Sciences, Department of Surgery, Sahlgrenska Academy, Gothenburg University, Gothenburg, Sweden; 2 Department of Development and Research, Armed Forces Center for Defense Medicine, Gothenburg, Västra Frölunda, Sweden; 3 Department of Aviation Security, Military University of Aviation, Dęblin, Poland; 4 Harvard Humanitarian Initiative, T.H. Chan School of Public Health, Harvard University, Boston, Massachusetts, USA

During public health emergencies and disasters, there are many threats to human health, which are related to the environmental elements, such as water, vectors, waste, and sanitation. In this regard, the access to clean water is so crucial for everyday domestic purposes that we take it for granted. Water is essential in establishing good sanitation (excreta disposal, vector control, solid and medical waste management and drainage), and hygienic measures such as water hygiene (eg, keeping water supplies safe), personal hygiene (eg, washing hands), domestic hygiene (eg, food hygiene) and environmental hygiene (eg, keeping household environments free of excreta and solid waste) [1,2].

In 1997, Webster described aquatic birds as reservoirs for all influenza A viruses, which could spread by fecal-oral transmission in untreated water. Influenza A viruses could further be transmitted to other species by the respiratory airborne route and become highly pathogenic. The transmission of avian influenza virus from their reservoirs to other species including humans could be prevented by treatment of the water supply and of avian protein sources with disinfectants or by heating [3]. The current coronavirus 2019 (COVID-19) is no exception since the survival of the large family of coronavirus in the water system has already been highlighted, which makes the water-based epidemiology mandatory to overcome the risk of virus spread [4]. A new report by Shutler et al. showed that COVID-19 could remain stable within water up to 25 days. Such a result could be devastating for countries with high infection rates since fecal contaminated rivers, waterways, and water systems can provide highly infectious doses implicating the need for freshwater systems and environmental interventions to prevent any virus resurgence [5].

## PUBLIC HEALTH AND PANDEMICS

Wastewater is a major social and environmental issue that has detrimental impacts on human health, economic productivity, the quality of ambient, freshwater, and the ecosystem, but may also be a crucial factor in spreading COVID-19 if left untreated [6,7]. To preserve the high quality and access to water, the same fundamental procedures of disaster and emergency prevention should be utilized. Public health engineering focuses on evaluating and managing environmental issues that have perceptible impacts on public health during disasters. Public health engineering professionals, managers, and responders have a critical function in managing environmental public health impacts during all crises [1]. For this reason, they need to collect data on the population, health, technical issues, physical environment, water transportation, water storage and distribution, water use, and relevant socio-political data (Assessment and analysis Phase) followed by strategic planning based on assessment and analysis. A global objective must be established, divided into specific objectives, which lead to outcomes/results. Activities to obtain each of these results must be determined. The manpower and logistics and a detailed budget needed for this process must also be defined [1]. Especially in some extreme disasters, quick implementation of the public health engineering plan is the definitive next step since it is not possible to obtain all the desired requirements without starting up an intervention. Indeed, the intervention and implementation phase should be monitored to guarantee the permanent appropriateness and create a match between objectives, implemented measures, expected results, and the outcome obtained within the time frame of the intervention. Finally, the evaluation is intended to look at the real impact of public health engineering interventions on the situation of relevant services such as safe drinking water and waste management, sanitation, and shelter, to name but a few [1,8].

## TRACKING THE UNTRACEABLE

In the heat of the current COVID-19 pandemic, initial interventions have been directed to the detection of cases, emergency treatment of the virus, and finding ways to cease the increasing number of deaths [9]. However, due to the lack of effective treatments and a vaccine, preventive measures such as individual protection and hygiene, and physical distancing have become crucial to every society. In some cases, long-standing quarantine has been implemented, resulting in temporary improvements [10]. Despite these efforts, COVID-19 has spread unimpeded continuously in many parts of the world and has now reentered isolated societies after implementing quarantine. Such a pattern may raise the question of whether our management system is adequately working, and if the pandemic has developed different patterns to ensure its survival? In a newly published paper, French scientists performed a time-course quantitative analysis of COVID-19 in raw wastewater samples collected from several major wastewater treatment plants (WWTPs) [11]. The sampling period included the lockdown period in France. According to the results obtained, the viral genome could be detected before the beginning of the exponential growth of the epidemic. Furthermore, the increase of genome units in raw wastewaters accurately followed the observed increase of human COVID-19 cases regionally. Similarly, the number of genome units decreased concomitantly with the reduction in the number of newly infected cases during the lockdown [11]. These results show that the virus can be traced and indicate a need for quantitative monitoring of COVID-19 genomes in wastewaters. Such additional information can be used to improve the survey of COVID-19 circulation, locally or regionally [11,12].

## LESSONS LEARNED – WE DO AS WE LEARN

Lessons learned and evidence-based management should be utilized when facing rarities, such as disasters and pandemics. Unfortunately, this is not the case. The current COVID-19 pandemic has deviated from these principles and strategies due to political and economic considerations, consequently endangering the life of all beings, resulting in many shortcomings [9-14]. In prosperous countries, the spread of the disease appears to be faster, causing more deaths and creating resource scarcity [10]. One reason might be the very tight infrastructure that enables the spread of disease through direct contact. Another reason, however, can be the possibility of releasing wastewater directly to the environment without any adequate treatment.

Although the most highly developed countries such as the US were supposed to be an exception to this phenomenon, the current COVID-19 pandemic has challenged established public health capabilities, suggesting that human activities using water produce wastewater and the resultant pollution loads are increasing worldwide [8,15]. In the USA, the number of infected cases and deaths are increasing daily. According to Honolulu Civil Beat, Hawaii has the fastest growing infection rate in the country compared to what was reported months ago [16]. One reason is the lack of federal guidance regarding parameters needed for virus control, such as wastewater plants control, which results in late multidisciplinary collaboration and wastewater testing [17]. The other might be the discrepancy in testing between two periods. However, there seems to be a lack of solid and united information to the public, which results in some people still not being convinced about the need of implementing the recommendations given by US public health officials. Correct and timely information, contact tracing, physical distancing, and public health engineering are all necessary parts of the public health management strategies that must be professionally and rapidly implemented [1,8,11-14].

**Figure:**
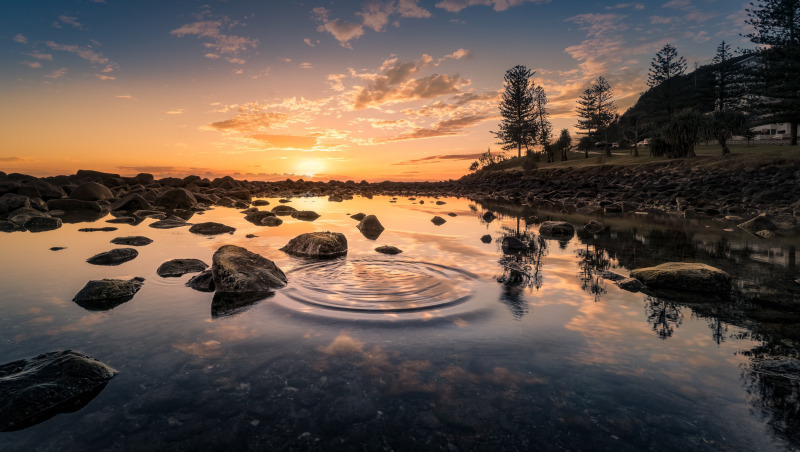
Photo: From https://pixabay.com/photos/landscape-lake-sunset-reflection-1802337/.

## CONCLUSIONS

It must be remembered that a defective water management system for drinking or personal use cannot only worsen the medical management of an emergency but can also contribute to spreading the disease or other water-borne conditions. The recent events in one of the most developed countries in the world represent a clear dismissal of scientific evidence for public health management and serves as a lesson for many [18].

Irrespective of the cause of an emergency or disaster, the response is a multidisciplinary task, which involves many actors. No health care system can work alone and without a secure, safe, and pure water resource.

Although wastewater can create additional difficulties, within the COVID-19 crisis, opportunities have emerged to apply innovative solutions. Among others, technological advancement in monitoring of socio-ecological conditions and immigrant tracks and travel volumes have all shown to be significant measures to limit the spread of the virus [19]. Furthermore, innovative technologies within three new topics: 1) Resource efficiency in treatment by improved aeration, modeling and energy audit, improved biogas production, etc.; 2) Facilitating water reuse, by online monitoring, efficient disinfection and treatment of pharmaceutical residues; and 3) Resource recovery, by sludge valorisation with recycling of nutrients and other valuables, have recently been demonstrated within the EU [20,21]. Resource efficiency in treatment by improved aeration, modelling and energy audit, improved biogas production, etc.; Facilitating water reuse, by online monitoring, efficient disinfection and treatment of pharmaceutical residues; and Resource recovery, by sludge valorisation with recycling of nutrients and other valuables, have recently been demonstrated within the EU [20]. Future policy and decision-making globally, should incorporate thorough after-action reports and government commissions to investigate best practices and lessons learned from the COVID-19 pandemic [9].
